# Seasonality of Influenza A(H3N2) Virus: A Hong Kong Perspective (1997–2006)

**DOI:** 10.1371/journal.pone.0002768

**Published:** 2008-07-23

**Authors:** Julian W. Tang, Karry L. K. Ngai, Wai Y. Lam, Paul K. S. Chan

**Affiliations:** 1 Department of Microbiology, The Chinese University of Hong Kong, Prince of Wales Hospital, Shatin, New Territories, Hong Kong Special Administrative Region, China; 2 Stanley Ho Centre for Emerging Infectious Diseases, The Chinese University of Hong Kong, Prince of Wales Hospital, Shatin, New Territories, Hong Kong Special Administrative Region, China; National Cancer Institute, United States of America

## Abstract

**Background:**

The underlying basis for the seasonality of influenza A viruses is still uncertain. Phylogenetic studies investigated this phenomenon but have lacked sequences from more subtropical and tropical regions, particularly from Southeast Asia.

**Methodology/Principal Findings:**

281 complete hemagglutinin (HA) and neuraminidase (NA) sequences were obtained from influenza A(H3N2) viruses, collected over 10 years (1997–2006) from Hong Kong. These dated sequences were analyzed with influenza A(H3N2) vaccine strain sequences (Syd/5/97, Mos/10/99, Fuj/411/02, Cal/7/04) and 315 other publicly available dated sequences from elsewhere, worldwide. In addition, the NA sequence alignment was inspected for the presence of any naturally occurring, known, neuraminidase inhibitor (NAI) resistance-associated amino acid mutations (R292K and E119V). Before 2001, the Hong Kong HA and NA sequences clustered more closely with the older vaccine sequences (Syd/5/97, Mos/10/99) than did sequences from elsewhere. After 2001, this trend reversed with significant clusters containing HA and NA sequences from different locations, isolated at different times, suggesting that viral migration may account for much of the influenza A(H3N2) seasonality during this 10-year period. However, at least one example from Hong Kong was found suggesting that in some years, influenza A(H3N2) viruses may persist in the same location, perhaps continuing to circulate, sub-clinically, at low levels between seasons, to re-emerge in the influenza season the following year, relatively unchanged. None of these Hong Kong influenza A(H3N2) NA sequences contained any of the known NAI-resistance associated mutations.

**Conclusions/Significance:**

The seasonality of influenza A(H3N2) may be largely due to global migration, with similar viruses appearing in different countries at different times. However, occasionally, some viruses may remain within a single location and continue to circulate within that population, to re-emerge during the next influenza season, with relatively little genetic change. Naturally occurring NAI resistance mutations were absent or, at least, very rare in this population.

## Introduction

Despite many hypotheses and studies, the underlying basis for the annual recurrence of seasonal influenza still remains a mystery [1]. Hammond et al. [2] postulated a rapid, global dispersion of ‘airborne aerosolized influenza virus’ via the atmosphere, to account for the persistence and spread of the disease. Recent reviews have discussed the various approaches to resolving this question, and identified various factors that may be involved, including: properties of the virus itself (mutation rates and immune escape), properties of the host (seasonal variation in host health and behavior, e.g. crowding and air travel, production and dissemination of bioaerosols through sneezing and coughing), and properties of the environment (temperature, humidity and weather variations, e.g. El Nino) [3]–[7].

Some of these factors have been incorporated into mathematical models to attempt to understand the driving forces behind influenza seasonality [6], [8]–[13].

Sequence-based analyses have become very popular recently and have shed some interesting insights into possible underlying mechanisms of influenza seasonality. Many of these also urge for (or at least hint at) the need for more sequences from tropical regions to be made publicly available to increase the accuracy of such analyses [14]–[21]. Other studies have analyzed genetic data together with the even more scarcely available antigenic data, in attempts to understand and even predict the most likely emerging strains [22]–[25]. Even the application of mass spectrometry has been applied to influenza surveillance [26].

Hong Kong is a subtropical region of almost 7 million people, 95% of whom are ethnic Chinese, with a mean temperature of 24°C and mean relative humidity of 79% [27]. It lies geographically in the Northern hemisphere, and its influenza season occurs during February–April, sometimes with a second peak during June–August, each year. In contrast, other Northern hemisphere countries usually have a more extended influenza season from November to March/April, whereas the influenza season of Southern hemisphere countries usually occur from May to September [7], [28]. Hence, Hong Kong may be unique in that its biphasic influenza seasonality seems to straddle those of the Northern and Southern hemisphere countries, making the molecular epidemiology of its circulating influenza viruses of great interest. In addition, Hong Kong and Southern China have been referred to as the ‘epicenter’ for new influenza A viruses with pandemic potential for over 25 years now [29]. For all of these reasons, any investigation of the underlying basis for influenza seasonality may benefit greatly from a study of influenza viruses isolated from Hong Kong.

In this study, an analysis is presented of 281 Hong Kong influenza A(H3N2) hemagglutinin (HA) and neuraminidase (NA) full-length, dated sequences collected over 10 years (1997–2006) to assist the ongoing efforts to elucidate the underlying basis for the seasonality of influenza A(H3N2).

## Results

The HA and NA ML phylogenetic trees (with and without the additional, down-loaded contemporary sequences from publicly available archives) produced in this study are too large to include as separate figures in this paper and have been published as online Supporting Information in a scrollable PDF format for further inspection on the PLoS ONE journal website (http://www.plosone.org/home.action).

For each of these trees, certain clusters of interest have been highlighted using annotated red boxes or ellipses, and will be specifically referred to, in the following text for further description and discussion.

All of these 281 Hong Kong influenza A(H3N2) HA and NA sequences have been deposited on GenBank (Accession nos.: EU856814-EU857094 for HA, and EU857095-EU857375 for NA sequences).

Figures S1 and S2 show the relationship between the 281 HA and NA sequences for the Hong Kong influenza A(H3N2) samples and 4 World Health Organization (WHO) influenza A(H3N2) vaccine strain HA sequences (Syd/5/97, Mos/10/99, Fuj/411/02 and Cal/7/04).

These Hong Kong HA and NA sequences were inspected to determine if there were any sequences from consecutive influenza seasons occurring on the same branch, indicating that viruses with the same or very similar HA and NA gene sequences were occurring in adjacent influenza seasons. This would suggest that that particular virus carrying this gene may have remained ‘latent’ in that population, to re-emerge in the same population the following season.

One example of such possible viral persistence between influenza seasons was found, with HA and NA sequences from the same viruses (5251Jan02 and 5267Jan03, as indicated in Figures S1 and S2 for the HA and NA phylogenetic trees, respectively), showing a similar clustering pattern for both these genes, separated by at least one year.

Interestingly, the HA sequence from sample 5250Jan02 clusters closely with those from samples 5251Jan02 and 5267Jan03 (Figures S1 and S3), but its NA sequence lies some distance away on a separate branch (Figures S2 and S4). This may suggest a possible reassortment event, either with its HA or NA gene segment. Further full genome sequencing and analysis may resolve this issue.

The red boxes in both Figures S1 and S2 highlight all the other January sequences (where available), for the other years during 1997–2006. It is interesting to note that before 2001, the Hong Kong HA and NA sequences cluster closely with the WHO vaccine strains Syd/5/97 and Mos/10/99, but after 2001, they are more scattered. No Hong Kong NA sequences cluster with the Fuj/411/02 and Cal/7/04 WHO vaccine HA sequences. Similarly, no Hong Kong NA sequences cluster with the Fuj/411/02 NA sequence, and only a few cluster with the Cal/7/04 WHO vaccine NA sequence.

The relationship between the WHO vaccine HA and NA sequences and those from Hong Kong and elsewhere can be seen even more clearly in Figures S3 and S4 when the 315 JCVI sequences are added to each tree. Although these contemporary JCVI sequences are mainly drawn from just three additional locations, they still represent the Northern hemisphere (New York, USA) and the Southern hemisphere (Western Australia and various locations in New Zealand). Again, for reference, the January Hong Kong HA and NA sequences from each year are again highlighted in red boxes.

In addition, in Figures S3 and S4, red ellipses have been added to show where similar HA and NA sequences from other, non-Hong Kong locations have clustered with Hong Kong sequences on the same branch. The dates of such sequences may be the same (within the limit of the one month temporal resolution used in this study), relatively similar, or very different. These highlighted clusters serve to demonstrate the mobility and ubiquity of this influenza A(H3N2) virus, worldwide, during this 10-year period, i.e. genetically similar viruses can appear in different parts of the world at similar and also different times. These examples are not meant to be exhaustive and other such examples may be found in these trees.

These number and position of the clusters indicated by the red ellipses differ between Figure S3 (HA sequences) and Figure S4 (NA sequences) probably because there are different selection pressures acting on these two genes as they have quite different functions (i.e. the HA protein is used by the virus to bind to the host cell for entry, whereas the NA protein is an enzyme that enables new progeny viruses to leave the host cell). Also, in Figures 3 and 4, for both the HA and NA gene sequences, respectively, there is a large region of transition between Mos/10/99-like and Fuj/411/02-like viruses, containing sequences collected during 1999–2005.

### N2 neuraminidase inhibitor (NAI)-resistance associated mutations

Inspection of the protein-coding alignment for NA sequences showed no evidence of any of the known N2 neuraminidase inhibitor (NAI) resistance-associated mutations, R292K and E119V, in any of these Hong Kong influenza A(H3N2) isolates collected between 1997–2006.

## Discussion

This comparative analysis of dated HA and NA sequences from influenza A(H3N2) viruses from 4 geographical regions of the world (New York, Western Australia, New Zealand and Hong Kong) attempts to elucidate more clearly the behavior of influenza A(H3N2). This study contributes an additional 10 years of influenza A(H3N2) HA and NA sequences, from Hong Kong, to the publicly available sequence database (GenBank), which should aid other researchers investigating an underlying basis for influenza A(H3N2) seasonality.

The approach of the analysis in this study has been to compare accurately dated HA and NA sequences using established phylogenetic techniques to examine which sets of sequences cluster together, and by examining the dates and locations from which they were collected, to infer the movements of the virus within those dated periods.

A similar analysis was recently performed using dated whole genome influenza A(H3N2) sequences from New York, New Zealand and Australia, downloaded from publicly available databases, in an attempt to test two competing hypotheses: whether seasonal influenza A(H3N2) viruses continuously ‘migrate’ around the world, particularly between Northern and Southern hemispheres; or whether the virus remains ‘latent’ in one location and reactivates each year to produce the familiar pattern of influenza seasonality [19]. As these authors stated, ideally, whole genomes should be used for more accurate phylogenetic analyses of influenza virus as has been reported previously [14], [15], with at least one good reason for this being the potentially misleading conclusions caused by influenza viral HA and NA gene reassortments [19]. However, many laboratories worldwide do not have the resources to perform whole genome sequencing, which is expensive – particularly those in subtropical and tropical regions from where such influenza sequence data is significantly lacking.

In addition, with any phylogenetic study such as this, there is always a limitation on the number of sequences that are available (i.e. the number of respiratory samples containing influenza viruses that have been collected and sequenced in any one influenza season), and the number that can be comfortably analyzed within a given time-frame (i.e. the limitations on computing power, which again may be more of a problem in tropical and subtropical countries that are more resource-limited).

In this particular study, there is also a problem of sample bias as these sequences were obtained from only hospitalized children (rather than from those who remained in the community), and may therefore reflect the influenza virus population isolated from the more clinically severely ill patients (or those with more concerned, anxious parents). However, there was a rationale for deliberately selecting children's samples for this study. The reason for this is that, especially in Hong Kong, unlike adults, children of school age (1–10 years old) are more likely to stay close to home and not travel far from home, which would minimize any importation of influenza viruses from overseas. This would therefore reduce this confounding factor when assessing the migration vs latency hypotheses as an explanation for the underlying mechanism of influenza seasonality in Hong Kong.

Accepting all of these shortcomings, some of which are inevitable for such phylogenetic studies (since not all samples can be collected and sequenced from all infected individuals from all over the world for any particular virus), there are still some useful conclusions that can be gained from this study:

i) from Figures S1 and S2, there is at least one example of a virus that reappears, relatively unchanged between consecutive influenza seasons, and which can be seen as some evidence to support the ‘latency’ hypothesis [19]. Since the same viruses show this same pattern of clustering in both their HA and NA genes, this reduces the likelihood that this was due to a reassortment event in one of these genes, i.e. it is less likely for the same viruses to have reassorted both the HA and NA genes during the same years – though of course this possibility cannot be ruled out. Whole genome sequencing would be useful to confirm this if resources are available in the future.
ii) from Figures S3 and S4, it is difficult to say whether viruses from Hong Kong preceded (or gave rise to) those from elsewhere, since viruses from outside Hong Kong can be found to both pre-date and post-date those isolated from Hong Kong. However, there are several examples of similar viruses being isolated in different parts of the world at about the same time (as shown in some of the red ellipses in Figures S3 and S4).
iii) from Figures S1 to S4, before 2001, it can be seen that the Hong Kong HA and NA sequences tend to cluster more closely with the Syd/5/97 and Mos/10/99 WHO vaccine sequences - particularly with the HA sequences and Mos/10/99. After 2001, HA and NA sequences from non-Hong Kong viruses cluster more closely with the Fuj/411/02 and Cal/7/04 WHO vaccine sequences, which may be just a reflection of the poor vaccine strain match during the emergence of the Fuj/411/02-like viruses during 2002–2004, with Hong Kong being one of the last regions to be invaded by this strain. Most of the HA and NA sequences clustering closely with the Fuji/411/02 and Cal/7/04 WHO vaccine strains seem to have been isolated in Australasia (Figures S3 and S4), though this may not necessarily mean that the Fuj/411/02-like viruses originated from there.

Hence, these results suggest that the seasonality of influenza A(H3N2) may, in fact, result from a combination of these 2 models postulated by Nelson et al [19], i.e. mainly migration, but with occasional examples of latency. Again, due to the unavoidable, incomplete sampling and sequencing of influenza A(H3N2) viruses worldwide, this support for these hypotheses (‘migration’ and ‘latency’), as presented here, is admittedly, only patchy at best. However, the fact that we can find at least one example supporting the latency hypothesis, in these 281 HA and NA sequences spanning 10 years (1997–2006), suggests that they may both play a part in the underlying mechanism governing influenza A(H3N2) seasonality.

The large variations in both the HA and NA sequences during the transitional period between Mos/10/99 and Fuj/411/02 (Figure S3 and S4) may have reduced the protective efficacy of any pre-existing influenza antibodies (from prior infection or immunization by Mos/10/99-like viruses) before the inclusion of the Fuj/411/02 strain in the World Health Organization (WHO) recommendations for the seasonal influenza vaccine in 2004. Such a reduction in protective efficacy from the contemporary WHO influenza vaccine has indeed been suggested in several reports [14], [30], [31]. Similarly, although, the NA antigen is not usually specified in seasonal influenza vaccine, and its protective effect is unknown, its sequence variation in NA during this transitional period, may have also contributed to the reduced protection provided by the seasonal influenza A(H3N2) vaccine component during this period, when the Fuj/411/02-like viruses were emerging.

**Figure 1:**
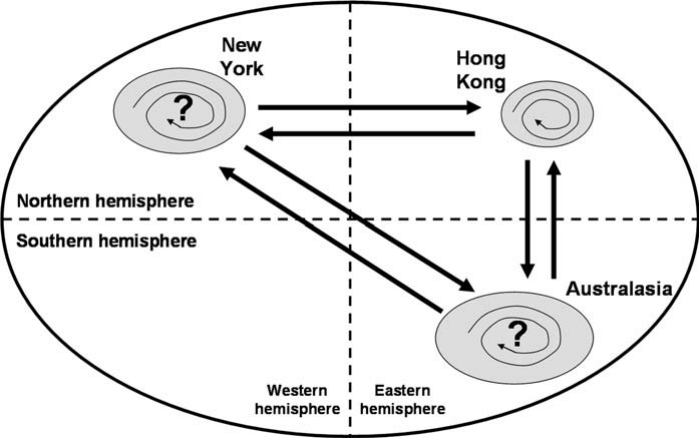
Summary. Influenza A(H3N2) seasonality may mainly result from ‘migration’ (thicker straight arrows) where different influenza A(H3N2) viruses move around the world between different populations at different times of the year. However, some influenza A(H3N2) viruses may remain and persist within the same population and location (thinner spiral arrows), circulating at low (perhaps subclinical) levels between influenza seasons (‘latency’), to re-emerge the following season with relatively little genetic change. The details in this figure are limited by the number and geographical locations of influenza sequence data available for analysis.

In summary, this study has provided additional data from Hong Kong to support a mainly migratory mechanism to explain the underlying seasonality of influenza A(H3N2) viruses. However, there may be small localities of so-called ‘latency’ where viruses remain circulating at low levels within that local population, to re-emerge during the influenza season of the following year, with relatively little genetic change. This may affect only a minority of these populations, with the majority being infected by newly imported influenza viruses from elsewhere. These concepts are summarized in Figure 1.

Two recent papers [37], [38] have suggested that the existing evidence tends to support a migration rather than a latency mechanism to explain the annual seasonality of influenza A. However, they did not have access to large numbers of influenza sequences from Southeast Asia. Thus, in view of the new data presented in this study, it is hoped that such hypotheses may be revised, to include a contribution from the latency mechanism. In some populations (perhaps those more localized in Southeast Asia), this latency mechanism may contribute more significantly to the underlying basis for the seasonality of influenza A – a possibility not entirely ruled out by one of these studies [38]. More data is required to explore this hypothesis in those populations. Admittedly, the fact that in this 10-year (1997–2006) data set of almost 300 influenza A(H3N2) HA and NA sequences from Hong Kong, we have only found one particular example supporting the latency mechanism, does suggest that this is a relative rarity, and that the migration mechanism is probably responsible for the majority of influenza seasonality patterns seen worldwide.

Finally, it is interesting, and to a certain extent, reassuring, to note that during this 10-year period (1997–2006), no influenza A(H3N2) viruses isolated from this cohort of NAI-naïve children from Hong Kong, showed any evidence of naturally occurring, known NAI resistance-associated amino acid mutations (R292K and E119V).

There are many parts of the puzzle remaining, such as exactly how do some of these influenza viruses migrate so widely, yet still remain relatively similar over several years. How is the low level circulation of ‘latent’ influenza viruses accomplished between seasons? Is this a property of the population's host immune responses, the virus, the environment or some combination of each of these factors?

In a collaborative effort to answer these questions and perhaps to improve the accuracy of influenza strain forecasting and vaccine composition [38], it is also hoped that this study will encourage more researchers in Southeast Asia to make their influenza sequences publicly available for analysis - especially whole genome sequences where resources permit, and particularly sequences from more tropical countries where influenza is prevalent all year round, with less well-defined seasonal peaks.

## Materials and Methods

### Source of influenza isolates

Approximately 30 influenza A(H3N2) isolates for each year of 1997–2006 obtained from the nasopharyngeal aspirates (NPAs) of children aged 1–10 years, admitted to the Prince of Wales Hospital (PWH) in Hong Kong, were retrieved from long-term archives (stored either at −70°C or in liquid nitrogen) for hemagglutinin (HA) and neuraminidase (NA) gene sequencing and analysis. These children presented with acute respiratory illness and did not receive anti-influenza therapy before or during their illness. As influenza A(H1N1) was the predominant circulating virus during 2006, very few H3N2 isolates were obtained for that year. Table 1 shows the age and sex distribution of these children.

**Table 1:** Source of influenza A(H3N2) isolates.

Year of collection	No. of solates	Age Mean (standard deviation) in years	Male∶Female
1997	30	1.6 (0.4)	17∶13
1998	30	2.8 (1.2)	24∶6
1999	30	2.3 (0.8)	21∶9
2000	30	2.7 (1.2)	17∶13
2001	29	2.4 (1.0)	18∶11
2002	30	2.5 (1.0)	11∶19
2003	30	2.7 (1.1)	15∶15
2004	30	3.5 (1.2)	18∶12
2005	30	3.1 (1.1)	15∶15
2006*	12	2.5 (0.8)	6∶6

* Influenza A(H1N1) became predominant during 2006 in this Hong Kong population, so fewer influenza A(H3N2) isolates were obtained during this year.

Verbal consent for the initial diagnostic testing of these samples for respiratory viruses, including influenza, was obtained from the parents of these children. Such verbal (rather than written) consent is routine for such standard diagnostic tests in Hong Kong. The local Joint Chinese University of Hong Kong-New Territories East Cluster (Joint CUHK-NTEC) Research Ethics Committee institutional review board awarded ethics approval for this retrospective sequencing and molecular epidemiological study (study reference number: CRE-2005.390) without the need to obtain further, explicit, written consent. This is also in agreement with the American College of Epidemiology guidelines [33] for such retrospective epidemiological/surveillance studies.

### HA and NA gene sequencing

The samples retrieved from the deep-freeze archive were first generation isolates, i.e. the original clinical samples (NPAs) had been inoculated, for routine diagnostic testing, into Madin-Darby Canine Kidney (MDCK) cells. These MDCK-cultured viral isolates were originally confirmed to be influenza A(H3N2) before being frozen and archived. For this study, these frozen isolates were retrieved from deep-freeze, thawed and then used directly for sequencing. If any of these newly-thawed, archived samples failed to amplify at this stage, as determined by ethidium bromide staining and gel electrophoresis, it was inoculated into MDCK cells and re-cultured. After this additional step, most isolates were successfully sequenced. If this step still failed, then an alternative isolate was retrieved from deep-freeze for sequencing.

Total RNA was extracted using the PureLink™ Viral RNA/DNA Kit (Invitrogen, Carlsbad, USA) according to the manufacturer's instructions, and resuspended in 50 µL of RNase-free water. Reverse transcription-polymerase chain reaction (RT-PCR) was carried out with SuperScript III One-Step RT-PCR System with Platinum T*aq* DNA Polymerase kit (Invitrogen, Carlsbad, USA) according to the manufacturer's protocols. In brief, a 25-µL reaction mix containing 0.5 µM of each forward and reverse primers and 10 µL of extracted RNA template were used for the RT-PCR. Sets of primers was designed to amplify the complete influenza A(H3N2) HA and NA genes (Table 2).

**Table 2:** Primers for the amplification of full-length influenza A(H3N2) HA (product size 1726 bp) and NA (1439 bp) genes.

Primer Name	Primer sequence (5′-3′)	Size	Tm (°C)
**HA gene**			
H3HA1F (forward)	AGCAAAAGCAGGGGATAATTCTA	23	53
H3HA1737R (reverse)	AATGCACTCAAATGCAAATGTTG	23	54
**NA gene**			
FLU-A-seqNAF1 (forward)	GGAGTGAAGATGAATCCAA	19	54
FLU-A-seqNAR1 (reverse)	GTAGAAACAAGGAGTTTTTTC	21	52

The RT reaction (55°C for 30 min) was followed by 94°C for 2 min and 40 cycles of PCR (94°C for 30 sec, 50°C for 30 sec, and 68°C for 1 min 45 sec, for each cycle) and a final extension at 68°C for 10 min.

The PCR products were purified by MicroSpin Sephacryl S-400 HR columns (Amersham Biosciences, UK). Sequencing reactions were performed using BigDye® Terminator v3.1 Cycle Sequencing Kits (Applied Biosystems, Foster City, USA) with 2.5 µL of template cDNA. For sequencing the HA and NA genes, the primers used are shown in Table 3.

**Table 3:** Primers for sequencing of full-length influenza A(H3N2) HA (product size 1726 bp) and NA (1439 bp) genes.

Primer Name	Primer sequence (5′-3′)	Size	Tm (°C)
**HA gene**			
H3HA1F	AGCAAAAGCAGGGGATAATTCTA	23	53
H3HAseq184F	TGACTAATGCTACTGAGCTGGTTC	24	52
H3HAseq1128F	TACGGTTTCAGGCATCAAAATT	22	53
H3HAseq1128R	AATTTTGATGCCTGAAACCGTA	22	53
H3HAseq1737R	AATGCACTCAAATGCAAATGTTG	23	54
**NA gene**			
H1H3NA347R	CATGACACATAAGGTTCTCTTGTCA	25	53
NA-884to904F	TGTRTNTGCAGRGAYAAYTGG	21	48
H1N3HA1021R	CCATGACCACCTTCTTCATTGTT	23	55

Sequencing reactions were performed on an Applied Biosystems 3130 ABI sequencer (ABI, Foster City, USA) and in both directions to cross-check the results. Alignments of nucleotides sequences were carried out using SeqScape v2.5 (Applied Biosystems, Foster City, USA).

### Sequence analysis

These Hong Kong influenza A(H3N2) HA and NA gene sequences were aligned and edited in BIOEDIT v.7.0.9.0 [32]. After alignment and manual editing, to enable all sequences (i.e. both the Hong Kong and JCVI reference sequences) to have the same final length for the construction for each of the phylogenetic trees shown in Figures S1 to S4, the sequence lengths were:

Figure S1 (HA sequences from Hong Kong and WHO only): 1538 bp;
Figure S2 (NA sequences from Hong Kong and WHO only): 1422 bp;
Figure S3 (HA sequences from Hong Kong, JCVI and WHO): 1538 bp;
Figure S4 (NA sequences from Hong Kong, JCVI and WHO): 1395 bp.

This was due to different HA and NA sequence lengths being available in the respective JCVI and WHO sequence data bases.

Phylogenetic tree construction was performed with PAUP* version 4.0b10 [34] by using a maximum likelihood (ML) approach, under an optimum model of evolution as determined by MODELTEST v3.7 [35]. Due to the large dataset and to reduce the time required for computation, optimal trees were searched for by using a nearest neighbor interchange (NNI) heuristic search strategy. Bootstrapping was performed within PAUP* and displayed using exported PDF files created using Figtree v1.0 (previously available from: http://tree.bio.ed.ac.uk/software/figtree/), which were subsequently annotated using Adobe Acrobat Professional 6.0.

The phylogenetic trees for these influenza A(H3N2) HA and NA sequences for the period 1997–2006 were rooted against the reference HA and NA sequences obtained from the influenza vaccine strain A/Syd/5/97(H3N2) downloaded from the Los Alamos National Laboratory (LANL) database (http://www.flu.lanl.gov/vaccine/) [36]. This strain was used as it was representative of the influenza A(H3N2) viruses circulating in 1997, i.e. at the start of this Hong Kong influenza A(H3N2) archive. Other available HA and NA sequences for other vaccine strains (Mos/10/99, Fuj/411/02, Cal/7/04) were also downloaded and included in all the phylogenetic trees.

For comparison with other contemporary influenza A(H3N2) sequences worldwide between 1997–2006, all publicly available (at the time of this analysis) dated HA and NA sequences from children of similar ages (0–16 years old – the upper range was extended to include more sequences), spanning this period were downloaded from the then TIGR (The Institute for Genomics Research) - now referred to as the J. Craig Venter Institute (JCVI) Influenza Genome Project Website influenza resource: http://msc.jcvi.org/infl_a_virus/status.shtml).

A total of 315 JCVI contemporary HA and NA sequences spanning this period (1997–2006, see online Table S1), together with 4 vaccine HA and NA sequences, were downloaded (from the LANL) for comparative analysis with the 281 Hong Kong influenza A(H3N2) HA and NA sequences (covering 10 years, 1997–2006).

### Presence/absence of established N2 neuraminidase inhibitor (NAI) resistance-associated mutations, R292K and E119V

Once the NA nucleic acid sequences were obtained, they were aligned, in-frame for protein coding, and converted to amino acids using the built-in function in BIOEDIT. The presence or absence of the established NAI resistance-associated mutations, R292K and E119V, was then determined by inspection of the resulting amino acid alignment, with reference to the influenza A/Syd/5/97 (H3N2) NA sequence, which was isolated before the licensing and widespread use of neuraminidase inhibitors that began after 1999/2000.

## Supporting Information

Figure S1 A bootstrapped maximum likelihood tree of the 281 hemagglutinin (HA) sequences from Hong Kong (1997–2006) with corresponding HA sequences from 4 WHO seasonal influenza vaccine strains (Syd/5/97, Mos/10/99, Fuj/411/02, Cal/7/04). A maximum likelihood phylogenetic tree of 285 hemagglutinin (HA) sequences (1538 bp), consisting of 281 from Hong Kong (collected during 1997–2006) and 4 WHO vaccine HA sequences (from Syd/5/97, Mos/10/99, Fuj/411/02, Cal/7/04, all yellow highlighted in red boxes), aligned and edited in BioEdit, constructed using PAUP* under an optimum model of evolution (a general reversible time model with a proportion of invariable sites I, and a gamma distributed rate of substitution G, i.e. GTR+I+G), as selected by MODELTEST under the Akaike Information Criteria and displayed using FigTree. The red boxes highlight sequences from January samples, where available, between 1997 and 2006. In particular, sequences 5250Jan02, 5251Jan02 and 5627Jan03 occur on the same branch, demonstrating the persistence of this virus between influenza seasons 2002 and 2003 in Hong Kong. Only bootstrap values greater than or equal to 70 are shown. The scale bar units are substitutions/site.
(0.07 MB PDF)

Figure S2 A bootstrapped maximum likelihood tree of the 281 neuraminidase (NA) sequences from Hong Kong (1997–2006) with corresponding NA sequences from 4 WHO seasonal influenza vaccine strains (Syd/5/97, Mos/10/99, Fuj/411/02, Cal/7/04). A maximum likelihood phylogenetic tree of 285 neuraminidase (NA) sequences (1422 bp), consisting of 281 from Hong Kong (collected during 1997–2006) and 4 WHO vaccine NA sequences (from Syd/5/97, Mos/10/99, Fuj/411/02, Cal/7/04, all yellow highlighted in red boxes), aligned and edited in BioEdit, constructed using PAUP* under an optimum model of evolution (a general reversible time model with a proportion of invariable sites I, and a gamma distributed rate of substitution G, i.e. GTR+I+G), as selected by MODELTEST under the Akaike Information Criteria and displayed using FigTree. The red boxes highlight sequences from January samples, where available, between 1997 and 2006. In particular, sequences 5251Jan02 and 5627Jan03 occur on the same branch (in the absence of a significant bootstrap difference), demonstrating the persistence of this virus between influenza seasons 2002 and 2003 in Hong Kong. Only bootstrap values greater than or equal to 70 are shown. The scale bar units are substitutions/site.
(0.07 MB DOC)

Figure S3 A bootstrapped maximum likelihood tree of the 281 neuraminidase (HA) sequences from Hong Kong (1997–2006) with 315 contemporary JCVI and the 4 WHO seasonal influenza vaccine HA sequences. A maximum likelihood phylogenetic tree of 600 hemagglutinin (HA) sequences (1538 bp), consisting of 281 from Hong Kong (collected during 1997–2006), 315 contemporary dated sequences downloaded from the JCVI website and 4 WHO vaccine sequences (from Syd/5/97, Mos/10/99, Fuj/411/02, Cal/7/04, all yellow highlighted in red boxes), aligned and edited in BioEdit, constructed using PAUP* under an optimum model of evolution (a general reversible time model with a proportion of invariable sites I, and a gamma distributed rate of substitution G, i.e. GTR+I+G), as selected by MODELTEST under the Akaike Information Criteria and displayed using FigTree. The red boxes highlight sequences from January samples, where available, between 1997 and 2006. In particular, sequences 5250Jan02, 5251Jan02 and 5627Jan03 occur on the same branch (in the absence of a significant bootstrap difference), demonstrating the persistence of this virus between influenza seasons 2002 and 2003 in Hong Kong. In addition, red ellipses show examples of where Hong Kong and non-Hong Kong sequences occur on the same branch, demonstrating how similar influenza viruses may be widely distributed, both spatially and temporally. These examples are not meant to be exhaustive and there may be others within the tree. Location abbreviations: WAu: Western Australia; NY: New York; Tw: Tairawhiti, Wk: Waikato, Cb: Canterbury, WCo: West Coast, Wel: Wellington, CC: Christchurch, Dun: Dunedin (all from New Zealand). Hong Kong sequences all start with numbers. Only bootstrap values greater than or equal to 70 are shown. The scale bar units are substitutions/site.
(0.11 MB DOC)

Figure S4 A bootstrapped maximum likelihood tree of the 281 neuraminidase (NA) sequences from Hong Kong (1997–2006) with 315 contemporary JCVI and the 4 WHO seasonal influenza vaccine NA sequences. A maximum likelihood phylogenetic tree of 600 neuraminidase (NA) sequences (1395 bp), consisting of 281 from Hong Kong (collected during 1997–2006), 315 contemporary dated sequences downloaded from the JCVI website and 4 WHO vaccine sequences (from Syd/5/97, Mos/10/99, Fuj/411/02, Cal/7/04, all yellow highlighted in red boxes), aligned and edited in BioEdit, constructed using PAUP* under an optimum model of evolution (a general reversible time model with a proportion of invariable sites I, and a gamma distributed rate of substitution G, i.e. GTR+I+G), as selected by MODELTEST under the Akaike Information Criteria and displayed using FigTree. The red boxes highlight sequences from January samples, where available, between 1997 and 2006. In particular, sequences 5251Jan02 and 5627Jan03 occur on the same branch (in the absence of a significant bootstrap difference), demonstrating the persistence of this virus between influenza seasons 2002 and 2003 in Hong Kong. In addition, red ellipses show examples of where Hong Kong and non-Hong Kong sequences occur on the same branch, demonstrating how similar influenza viruses may be widely distributed, both spatially and temporally. These examples are not meant to be exhaustive and there may be others within the tree. Location abbreviations: WAu: Western Australia; NY: New York; Tw: Tairawhiti, Wk: Waikato, Cb: Canterbury, WCo: West Coast, Wel: Wellington, CC: Christchurch, Dun: Dunedin (all from New Zealand). Hong Kong sequences all start with numbers. Only bootstrap values greater than or equal to 70 are shown. The scale bar units are substitutions/site.
(0.11 MB DOC)

Table S1 The 315 downloaded TIGR(JCVI) influenza A(H3N2) HA and NA sequences used to construct the multi-country HA and NA phylogenetic trees shown in Figures S3 and S4. MS Excel file containing the GenBank Accession numbers of the 315 downloaded TIGR(JCVI) influenza A(H3N2) HA and NA sequences which were used to construct the multi-country HA and NA phylogenetic trees shown in Figures S3 and S4.
(0.94 MB DOC)
