# Supplementary figures and images for: Seasonality of Influenza A(H3N2) Virus: A Hong Kong Perspective (1997–2006)
Source: PLoS One. 2008 Jul 23;3(7):e2768. doi: 10.1371/journal.pone.0002768 (PMC2481298; doi:10.1371/journal.pone.0002768)

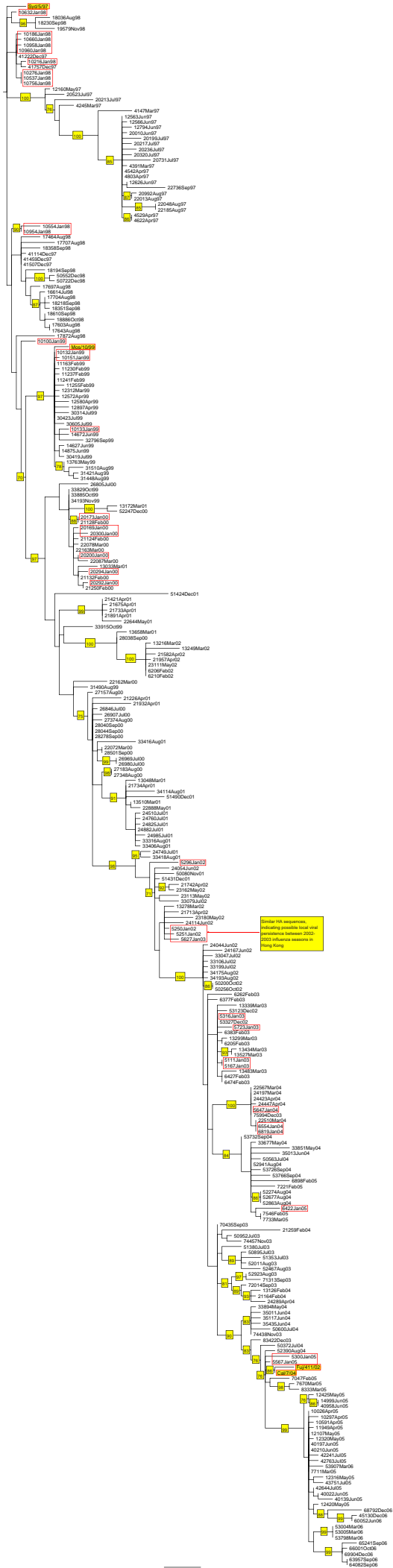

Supplement: Figure S1 — A bootstrapped maximum likelihood tree of the 281 hemagglutinin (HA) sequences from Hong Kong (1997–2006) with corresponding HA sequences from 4 WHO seasonal influenza vaccine strains (Syd/5/97, Mos/10/99, Fuj/411/02, Cal/7/04). A maximum likelihood phylogenetic tree of 285 hemagglutinin (HA) sequences (1538 bp), consisting of 281 from Hong Kong (collected during 1997–2006) and 4 WHO vaccine HA sequences (from Syd/5/97, Mos/10/99, Fuj/411/02, Cal/7/04, all yellow highlighted in red boxes), aligned and edited in BioEdit, constructed using PAUP* under an optimum model of evolution (a general reversible time model with a proportion of invariable sites I, and a gamma distributed rate of substitution G, i.e. GTR+I+G), as selected by MODELTEST under the Akaike Information Criteria and displayed using FigTree. The red boxes highlight sequences from January samples, where available, between 1997 and 2006. In particular, sequences 5250Jan02, 5251Jan02 and 5627Jan03 occur on the same branch, demonstrating the persistence of this virus between influenza seasons 2002 and 2003 in Hong Kong. Only bootstrap values greater than or equal to 70 are shown. The scale bar units are substitutions/site. (0.07 MB PDF) [file pone.0002768.s001.pdf]

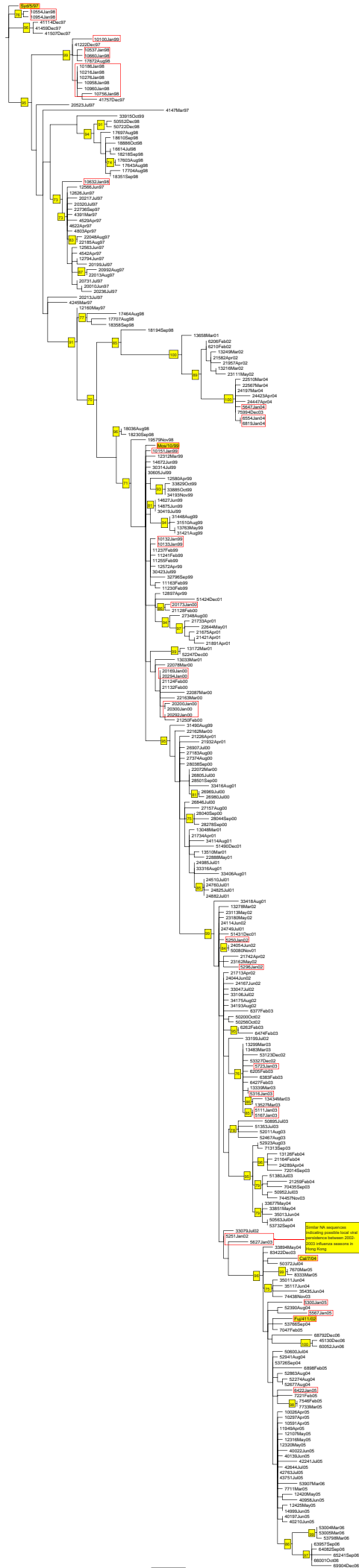

Supplement: Figure S2 — A bootstrapped maximum likelihood tree of the 281 neuraminidase (NA) sequences from Hong Kong (1997–2006) with corresponding NA sequences from 4 WHO seasonal influenza vaccine strains (Syd/5/97, Mos/10/99, Fuj/411/02, Cal/7/04). A maximum likelihood phylogenetic tree of 285 neuraminidase (NA) sequences (1422 bp), consisting of 281 from Hong Kong (collected during 1997–2006) and 4 WHO vaccine NA sequences (from Syd/5/97, Mos/10/99, Fuj/411/02, Cal/7/04, all yellow highlighted in red boxes), aligned and edited in BioEdit, constructed using PAUP* under an optimum model of evolution (a general reversible time model with a proportion of invariable sites I, and a gamma distributed rate of substitution G, i.e. GTR+I+G), as selected by MODELTEST under the Akaike Information Criteria and displayed using FigTree. The red boxes highlight sequences from January samples, where available, between 1997 and 2006. In particular, sequences 5251Jan02 and 5627Jan03 occur on the same branch (in the absence of a significant bootstrap difference), demonstrating the persistence of this virus between influenza seasons 2002 and 2003 in Hong Kong. Only bootstrap values greater than or equal to 70 are shown. The scale bar units are substitutions/site. (0.07 MB DOC) [file pone.0002768.s002.pdf]

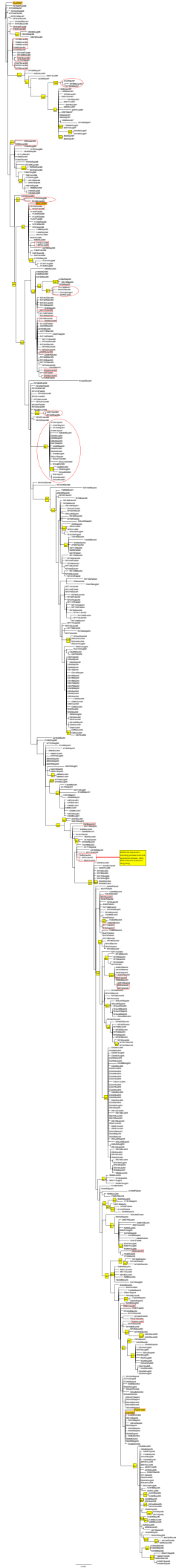

Supplement: Figure S3 — A bootstrapped maximum likelihood tree of the 281 neuraminidase (HA) sequences from Hong Kong (1997–2006) with 315 contemporary JCVI and the 4 WHO seasonal influenza vaccine HA sequences. A maximum likelihood phylogenetic tree of 600 hemagglutinin (HA) sequences (1538 bp), consisting of 281 from Hong Kong (collected during 1997–2006), 315 contemporary dated sequences downloaded from the JCVI website and 4 WHO vaccine sequences (from Syd/5/97, Mos/10/99, Fuj/411/02, Cal/7/04, all yellow highlighted in red boxes), aligned and edited in BioEdit, constructed using PAUP* under an optimum model of evolution (a general reversible time model with a proportion of invariable sites I, and a gamma distributed rate of substitution G, i.e. GTR+I+G), as selected by MODELTEST under the Akaike Information Criteria and displayed using FigTree. The red boxes highlight sequences from January samples, where available, between 1997 and 2006. In particular, sequences 5250Jan02, 5251Jan02 and 5627Jan03 occur on the same branch (in the absence of a significant bootstrap difference), demonstrating the persistence of this virus between influenza seasons 2002 and 2003 in Hong Kong. In addition, red ellipses show examples of where Hong Kong and non-Hong Kong sequences occur on the same branch, demonstrating how similar influenza viruses may be widely distributed, both spatially and temporally. These examples are not meant to be exhaustive and there may be others within the tree. Location abbreviations: WAu: Western Australia; NY: New York; Tw: Tairawhiti, Wk: Waikato, Cb: Canterbury, WCo: West Coast, Wel: Wellington, CC: Christchurch, Dun: Dunedin (all from New Zealand). Hong Kong sequences all start with numbers. Only bootstrap values greater than or equal to 70 are shown. The scale bar units are substitutions/site. (0.11 MB DOC) [file pone.0002768.s003.pdf]

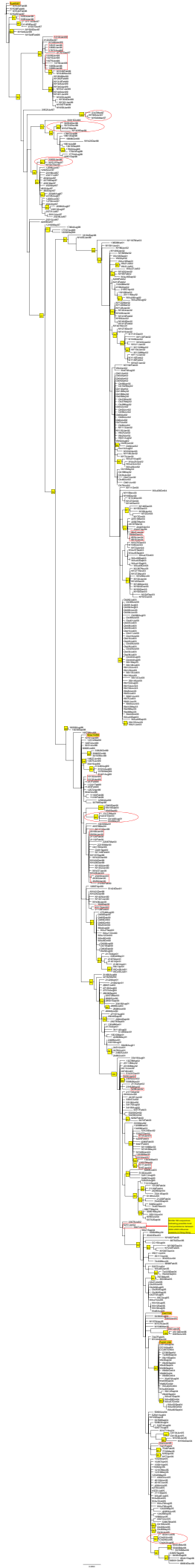

Supplement: Figure S4 — A bootstrapped maximum likelihood tree of the 281 neuraminidase (NA) sequences from Hong Kong (1997–2006) with 315 contemporary JCVI and the 4 WHO seasonal influenza vaccine NA sequences. A maximum likelihood phylogenetic tree of 600 neuraminidase (NA) sequences (1395 bp), consisting of 281 from Hong Kong (collected during 1997–2006), 315 contemporary dated sequences downloaded from the JCVI website and 4 WHO vaccine sequences (from Syd/5/97, Mos/10/99, Fuj/411/02, Cal/7/04, all yellow highlighted in red boxes), aligned and edited in BioEdit, constructed using PAUP* under an optimum model of evolution (a general reversible time model with a proportion of invariable sites I, and a gamma distributed rate of substitution G, i.e. GTR+I+G), as selected by MODELTEST under the Akaike Information Criteria and displayed using FigTree. The red boxes highlight sequences from January samples, where available, between 1997 and 2006. In particular, sequences 5251Jan02 and 5627Jan03 occur on the same branch (in the absence of a significant bootstrap difference), demonstrating the persistence of this virus between influenza seasons 2002 and 2003 in Hong Kong. In addition, red ellipses show examples of where Hong Kong and non-Hong Kong sequences occur on the same branch, demonstrating how similar influenza viruses may be widely distributed, both spatially and temporally. These examples are not meant to be exhaustive and there may be others within the tree. Location abbreviations: WAu: Western Australia; NY: New York; Tw: Tairawhiti, Wk: Waikato, Cb: Canterbury, WCo: West Coast, Wel: Wellington, CC: Christchurch, Dun: Dunedin (all from New Zealand). Hong Kong sequences all start with numbers. Only bootstrap values greater than or equal to 70 are shown. The scale bar units are substitutions/site. (0.11 MB DOC) [file pone.0002768.s004.pdf]
